# Endovascular Treatment of Extracranial Arteriovenous Malformations: A Retrospective Monocentric Case-Series Study

**DOI:** 10.3390/tomography11070075

**Published:** 2025-06-26

**Authors:** Giuseppe Sarti, Giovanni Barbato, Francesco Tiralongo, Gianpaolo Santini, Francesco Arienzo, Davide Nilo, Fabio Tortora, Alfonso Reginelli, Rosita Comune, Maria Borrelli, Stefania Tamburrini, Antonio Basile, Mariano Scaglione

**Affiliations:** 1 Department of Advanced Biomedical Sciences, University “Federico II”, Via Pansini, 5, 80131 Naples, Italy; giuseppe.sarti@unina.it (G.S.); fabio.tortora@unina.it (F.T.); 2 Department of Interventional Radiology, Ospedale del Mare ASL Napoli1 Centro, 80142 Naples, Italy; santinigianpaolo@libero.it (G.S.); francescoarienzo@virgilio.it (F.A.); 3 Radiology Unit 1, Department of Medical Surgical Sciences and Advanced Technologies “GF Ingrassia”, University Hospital Policlinico “G. Rodolico-San Marco”, University of Catania, 95124 Catania, Italy; basile.antonello73@gmail.com; 4 Department of Advanced Medical and Surgical Sciences, University of Campania “Luigi Vanvitelli”, 80138 Naples, Italy; nilodavide@gmail.com; 5 Department of Precision Medicine, University of Campania “Luigi Vanvitelli”, 80138 Naples, Italy; alfonso.reginelli@unicampania.it; 6 Department of Radiology, Ospedale del Mare ASL Napoli1 Centro, 80142 Naples, Italy; ros.comune@gmail.com (R.C.); borrellix@libero.it (M.B.); tamburrinistefania@gmail.com (S.T.); 7 Radiology Department of Surgery, Medicine and Pharmacy, University of Sassari, Viale S. Pietro, 07100 Sassari, Italy; mscaglione@uniss.it; 8 Department of Radiology, James Cook University Hospital & Teesside University, Marton Road Marton Rd., Middlesbrough TS4 3BW, UK

**Keywords:** arteriovenous malformation, embolization, endovascular treatment, Lauromacrogol, high-flow vascular malformation

## Abstract

Background: Extracranial arteriovenous malformations (AVMs) are rare congenital vascular anomalies that often require endovascular treatment due to symptoms such as pain, bleeding, or functional impairment. Endovascular strategies include arterial, venous, or combined embolization approaches; however, recurrence remains a major challenge. We retrospectively evaluate the technical success, safety, and clinical outcomes of arterial-only versus combined arterial and venous embolization for the treatment of extracranial AVMs. Materials and Methods: This single-center retrospective study included 14 patients (mean age 31.8 ± 21.7 years; 64% female) with symptomatic extracranial AVMs (Schobinger stage II) treated between 2017 and 2023. AVMs were classified angiographically (Yakes classification) and treated with embolization via arterial or combined access routes. The primary endpoint was technical success (defined as angiographic nidus occlusion), while secondary endpoints included clinical recurrence and procedure-related complications. Follow-up included clinical and Doppler ultrasound assessments. Results: Nine patients (64%) underwent arterial embolization alone; five (36%) received combined arterial and venous embolization, including Lauromacrogol injection via direct puncture. Technical success was achieved in all cases (100%). Clinical recurrence occurred in two patients (14%), both from the arterial-only group. One major complication (tongue ischemia) occurred in a single patient (7%). No complications or recurrences were observed in the combined treatment group. Statistical analysis showed no significant difference in recurrence or complication rates between groups.

## 1. Introduction

Extracranial arteriovenous malformations (AVMs) are rare congenital vascular anomalies characterized by direct communications between arteries and veins that bypass the capillary bed. The anatomical absence of a capillary network in the AVM nidus results in high-flow arteriovenous shunting through one or more fistulas [1], which tend to expand over time. AVMs are congenital conditions that persist throughout life and never regress spontaneously [2,3].

While the true prevalence of AVMs remains uncertain due to underdiagnosis and the heterogeneous clinical spectrum, extracranial AVMs account for approximately 10–15% of all vascular malformations seen in specialized referral centers [4]. Peripheral AVMs most frequently involve the head and neck region (up to 47% of cases), followed by the extremities (29%) and the trunk (11%) [5].

The pathogenesis of AVMs is not fully understood, though genetic defects and pro-angiogenic factors may play a role [6,7].

Although usually present at birth, these lesions often remain clinically silent until adolescence and may progress under the influence of hormonal changes, trauma, or surgical manipulation [4]. The natural history is characterized by a progressive course: children with early-stage lesions (Schobinger stage I) have shown an 83% risk of progression to more advanced stages before adulthood [5], emphasizing the need for timely and effective treatment.

AVMs can be classified as simple, often sporadic and isolated, or combined when two or more malformations coexist [8,9,10].

AVMs can be either focal or diffuse. Focal AVMs appear as soft tissue masses and are typically diagnosed during infancy or childhood. They may be associated with local erythema, hyperthermia, prominent pulsations, a palpable thrill, and an audible bruit [11]. These lesions have one or multiple arterial feeders, well-defined borders, and may or may not include a nidus. They generally respond well to appropriate treatment. Conversely, diffuse AVMs exhibit more aggressive behavior, often infiltrating and destroying adjacent tissues. The arteriovenous shunting characteristic of diffuse AVMs can significantly impact hemodynamics, leading to reduced capillary oxygenation, ischemia, local hypervascularity, vascular steal phenomenon, and increased venous pressure. These factors contribute to the destruction of soft and bony tissues, pain, ulceration, gangrene, severe hemorrhage, esthetic deformities, and functional impairments when the malformation reaches a significant size. Although uncommon, congestive heart failure is a possible complication of AVMs. Diffuse AVMs are more frequently diagnosed in older children and adults.

In recent decades, endovascular embolization has emerged as the preferred therapeutic approach for extracranial AVMs, given its minimally invasive nature and repeatability. Several embolic agents have been adopted for this purpose, including n-butyl cyanoacrylate (NBCA), Onyx, and absolute ethanol. Among these, ethanol is considered the only curative liquid agent due to its ability to induce endothelial destruction, although it is associated with significant toxicity and procedural risks. NBCA and Onyx, while effective in achieving devascularization, are not cytotoxic and may be associated with higher recurrence rates. More recently, Lauromacrogol, a sclerosant with endothelial-injuring properties and a favorable safety profile, has been proposed as an alternative, particularly in fragile or superficial lesions [12].

Despite the increasing use of embolic agents, the optimal approach for AVM management remains controversial. Most published studies focus on either trans-arterial or transvenous strategies, and comparative data on a combined arterial and venous approach are scarce. In addition, little evidence is available regarding the specific use of Lauromacrogol in this context, especially for complex AVMs such as Yakes type III lesions, which often require multilevel targeting of both inflow and outflow circuits [5].

The aim of this study was to retrospectively assess the safety, technical success, and clinical outcomes of endovascular treatment for extracranial AVMs.

## 2. Materials and Methods

### 2.1. Study Design

This was a single-center, retrospective study approved by the local ethics committee. Informed consent was obtained from all patients. We retrospectively reviewed all consecutive patients treated with endovascular embolization for extracranial AVMs between January 2017 and October 2023. Patients were identified through the patient record system at the Department of Interventional Radiology.

Inclusion criteria were: (a) diagnosis of extracranial AVM confirmed by imaging (CE-CT and/or DSA), (b) symptomatic lesions requiring treatment (pain, bleeding, cosmetic deformity, functional impairment), and (c) treatment with either arterial, venous, or combined embolization, and d) patients in class II of Schobinger classification system.

Exclusion criteria included: (a) follow-up shorter than 6 months, and (b) intracranial or spinal AVMs.

### 2.2. Imaging Evaluation and Classification

Preprocedural imaging included Doppler ultrasound, CE-CT, and digital subtraction angiography (DSA) based on AVM location and the age of patients. AVMs were classified according to the Yakes angiographic classification, which is based on the type of shunt and the morphology of the nidus. Each lesion was also clinically staged using the Schobinger classification. All cases were re-evaluated by two interventional radiologists with over 10 years of experience, blinded to the therapeutic approach performed. Cross-sectional imaging was used to assess soft tissue involvement, flow characteristics, and lesion extension. Follow-up imaging consisted of clinical examination and Doppler ultrasound at 3 and 6 months, and thereafter every 12 months.

### 2.3. Treatment Protocol and Embolization Strategy

Experienced interventional radiologists performed all procedures under conscious sedation or general anesthesia. The choice of embolization route—arterial, venous, or combined—was based on the angioarchitecture observed at baseline angiography and guided by the following decision-making algorithm:

- Arterial embolization was preferred in lesions with limited and accessible feeders.
- Venous embolization was favored when the dominant outflow vein (DOV) was accessible and formed the site of arteriovenous shunting.
- Combined arterial and venous embolization was used in complex lesions with multilevel shunting, high-flow recurrence, or partial response to single-route embolization.

### 2.4. Devices for AVM Embolization

#### 2.4.1. Arterial Side

Coils: Embolizing coils are composed of various materials, including steel, platinum, and gold, and may be bare or fibered with materials such as nylon, Dacron, polyester, silk, or wool to enhance thrombogenicity [13]. Available in different shapes and sizes, coils are often used in conjunction with other embolic agents. They may be free-release or controlled-release, with the latter preferred for AVMs due to its superior handling and embolization efficiency. When deployed, coils are compacted within the vessel using the catheter tip to form a stable plug [14]. Coils are commonly employed in large-caliber vessels due to high-flow components or fistulous portions of the AVM nidus. They may also be used prior to the administration of liquid embolic agents, such as EVOH (in our caseOnyx, Medtronic, Minneapolis, MN, USA), to reduce flow and facilitate plug formation in dominant feeding branches [15,16,17].

EVOH (Ethylene Vinyl Alcohol Copolymer): EVOH, specifically Onyx (Medtronic, Minneapolis, MN, USA), is a widely used embolic fluid in interventional radiology. It comprises ethylene vinyl alcohol copolymer (48 mol/L ethylene and 52 mol/L vinyl alcohol) dissolved in dimethyl sulfoxide (DMSO) and mixed with micronized tantalum powder for radiopacity. Onyx is available in two formulations: low-density (Onyx 18 to 34, containing 6–8% EVOH) and high-density (Onyx 500+, containing 20% EVOH) [18].

Polyvinyl Alcohol (PVA) Particles: At our institution, Contour particles (Contour PVA–Boston Scientific 250–355, 355–500, 500–710 microns) are used. PVA particles occlude vessels by adhering to the walls, triggering an inflammatory reaction that leads to fibrotic closure. However, they may occlude vessels more proximally than anticipated and risk causing unintended embolization of non-target vessels due to catheter occlusion [19].

Fibrin Glue: Fibrin glue consists of a solution of fibrinogen and thrombin, which coagulates instantly upon mixing. It is used in various interventional procedures as an embolic agent. The contrast-to-glue ratio varies depending on vessel diameter [20]. A key disadvantage is the difficulty in controlling delivery, increasing the risk of non-target embolization.

#### 2.4.2. Venous Side

Lauromacrogol: Lauromacrogol is a synthetic alcohol composed of 95% hydroxyl polyoxdodecane and 5% ethanol. It is widely used as a sclerosing agent for varicose veins and, more recently, in the treatment of AVMs, particularly those with a significant venous component. Lauromacrogol induces endothelial damage upon direct injection, triggering intravascular thrombosis that subsequently leads to vessel fibrosis and definitive occlusion [21].

### 2.5. Variables and Outcome Measures

The following variables were collected for each patient: age, sex, anatomical location of the AVM, and angiographic classification (Yakes type). Procedural details included the vascular access approach (arterial, venous, or combined), type and quantity of embolic agents used, and number of treatment sessions.

The primary outcome was technical success, defined as angiographic occlusion of the nidus at the end of the embolization procedure.

Secondary outcomes included clinical recurrence (defined as reappearance of AVM-related symptoms or imaging evidence of residual or recurrent AVM during follow-up), number of embolization sessions required, and occurrence of peri-procedural complications. Procedure-related complications, categorized as minor or major, were assessed using the CIRSE classification system, which ranges from grade 1 (an intra-procedural event managed during the same session without additional treatment, post-procedural consequences, or deviation from the expected clinical course) to grade 6 (death) [22].

### 2.6. Statistical Analysis

Data were analyzed using SPSS software (IBM SPSS Statistics version 20, SPSS Inc., Chicago, IL, USA). Categorical variables were expressed as frequencies and percentages; continuous variables were reported as mean ± standard deviation. Fisher’s exact test was used to assess outcome rates between patients treated with arterial versus combined approaches. A *p*-value < 0.05 was considered statistically significant.

## 3. Results

Fourteen patients (nine women [64%] and five men [36%]) were included in the study. The mean age was 31.8 ± 21.7 years (Table 1).

The most common clinical presentation was a pulsatile mass (n = 10 [71%]), followed by asymptomatic presentation (n = 3 [21%]) and pelvic pain (n = 1 [7%]). Imaging modalities used for diagnosis included computed tomography (CT) in nine patients (64%), ultrasonography in four (29%), and a combination of CT and ultrasonography in one (7%).

The most frequent anatomical location of the arteriovenous malformations (AVMs) was the lower extremity (n = 10 [71%]), followed by the neck (n = 2 [14%]), pelvis (n = 1 [7%]), and upper extremity (n = 1 [7%]). According to angiographic classification, nine patients (64%) had Type I AVMs and five (36%) had Type IV AVMs (Table 1).

Angiography was performed via right femoral arterial access in nine cases (64%) and via combined direct venous and right femoral arterial access in five cases (36%).

On the arterial side, the most common embolic agents were coils alone (n = 4 [29%]) and coils plus polyvinyl alcohol particles (n = 4 [29%]); other combinations are detailed in Table 2. On the venous side, lauromacrogol was used exclusively in all five treated cases. Overall, nine patients (64%) received arterial embolization only, and five patients (36%) underwent combined arterial and venous embolization.

Eight patients (57%) underwent a single-stage embolization procedure, whereas six (43%) with a large arterial component were treated in a planned two-stage protocol.

Technical success was achieved in all cases (100%). Clinical recurrence occurred in two patients (14%); in both cases, it was at the 1-month follow-up visit after the procedure. There were no complications related to the puncture site. One patient (7%) with a facial AVM involving the lingual branch experienced a major complication, with ischemic injury affecting two-thirds of the tongue; this resolved fully after appropriate odontostomatological management.

### Subgroup Analysis

Of the nine patients with Type I arteriovenous malformations managed solely by arterial embolization, lesions were located in the lower extremity in five patients (56%), in the neck in two (22%), in the pelvis in one (11%), and in the upper extremity in one (11%).

**Figure 1:**
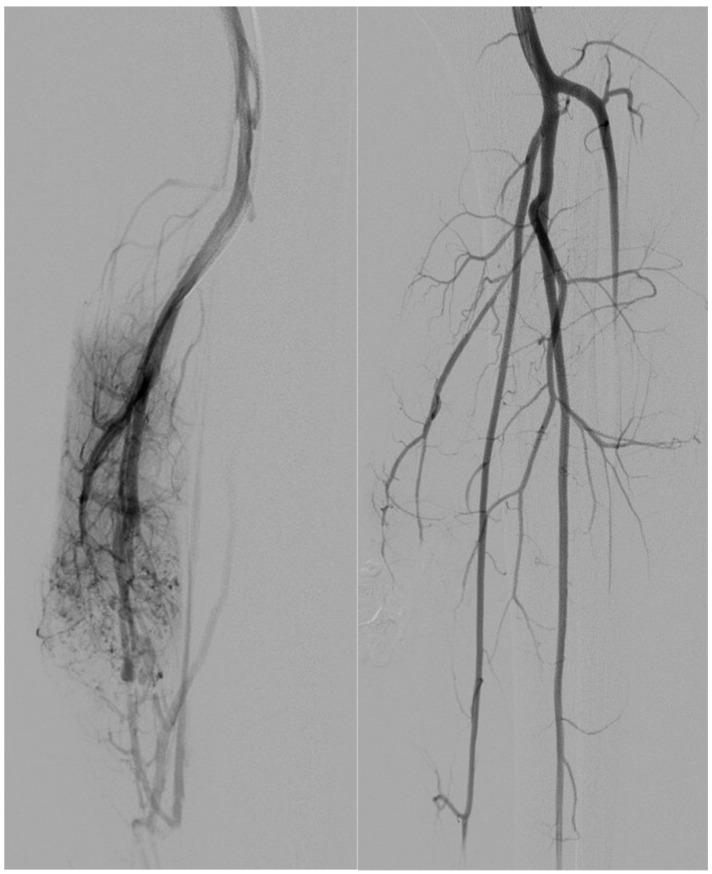
A 17-year-old patient. Type I AVM from the collateral branch of the peroneal artery. **The left image** shows an angiographic examination performed pre-procedure. **The right image** shows post-embolization angiography performed with a Progreat microcatheter and injecting EVOH particles alone.

Embolic regimens comprised a combination of coils and ethylene vinyl alcohol copolymer (EVOH) in four patients (44%), coils alone in three (33%), EVOH alone in one (11%) (Figure 1), and EVOH plus polyvinyl alcohol particles in one (11%).

Five patients (56%) underwent a single-stage embolization. Among the four who required a planned two-stage protocol, three received coils in the first session, followed by polyvinyl alcohol particles in two cases and additional coils in one; the remaining patient was treated initially with EVOH and subsequently with PVA.

Technical success was achieved in all cases. Clinical recurrence occurred in two patients (22%): one after single-stage EVOH embolization and one following the two-stage approach. One patient (11%) experienced a procedure-related complication.

Five patients with Type IV arteriovenous malformations underwent both arterial and venous embolization; all lesions were located in the lower extremity.

**Figure 2:**
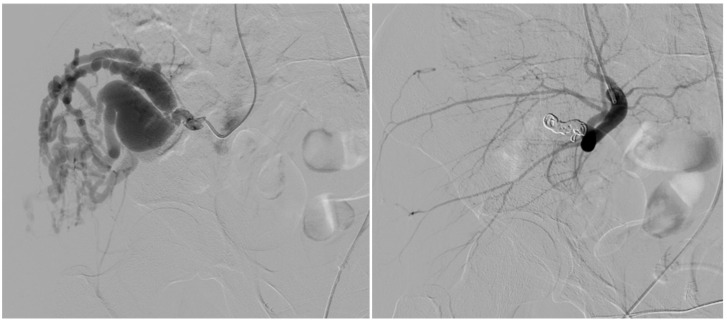
A 31-year-old patient presents with a Type IV AVM from the collateral branch of the gluteal artery. **The left image** shows an angiographic examination performed pre-procedure after super-selective catheterization of the vessel using a Progreat microcatheter. The image on the **right** shows post-embolization angiography performed using 4 mm coils.

**Figure 3:**
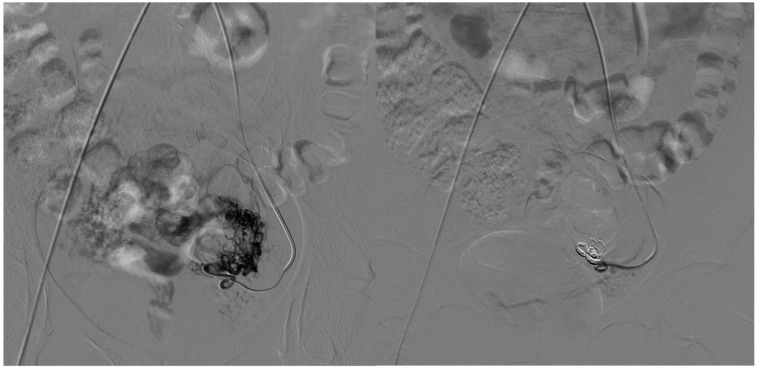
A 33-year-old patient presents with a Type I AVM from the collateral branch of the uterine artery. **The left image** shows an angiographic examination performed pre-procedure after super-selective catheterization of the vessel using an RUC catheter and a Progreat microcatheter. The image on the **right** shows post-embolization angiography performed using three 6 mm Nester coils and injecting EVOH.

On the arterial side, embolic materials were distributed as follows: coils alone in one patient (20%) (Figure 2), a combination of coils and ethylene vinyl alcohol copolymer (EVOH) in one (20%) (Figure 3), EVOH alone in one (20%), and polyvinyl alcohol particles (PVA) alone in one (20%).

Three patients (60%) underwent a single-stage procedure. Of the two patients treated in two stages, the first session comprised arterial coil embolization; the second session included arterial EVOH embolization combined with venous lauromacrogol injection.

Technical success was achieved in all cases. Neither clinical recurrence nor procedure-related complications were observed in this subgroup (0%).

Fisher’s exact test showed no statistically significant differences in recurrence rates (*p* = 0.505) and complications (*p* = 1) between the two groups.

## 4. Discussion

AVMs are a rare condition that should be differentiated from other vascular and non-vascular lesions based on their clinical and radiological features [8,10]. Various classification systems for AVMs exist, playing a crucial role in selecting individualized therapy and determining prognosis [8,9,23]. Several classifications have been proposed based on size, location, the specific blood vessel involved, or growth pattern. The first classification, proposed by Mulliken and Glowacki in 1982 [24], was based on endothelial characteristics. They classified vascular lesions into hemangiomas and vascular malformations (VMs). However, this classification has limitations, as hemangiomas and VMs have different etiologies and clinical features. Furthermore, hemangiomas are vascular tumors characterized by endothelial hyperplasia and rapid cell proliferation, typically absent at birth but developing within the first year of life.

The Schobinger classification (1990) categorized AVMs according to their growth pattern: quiescent (stage I), active or palpable growth (stage II), soft tissue destruction (stage III), and heart failure (stage IV). Due to the variability of the disease, Richter and Suen proposed modifications to include the number of cervicofacial subunits involved and the depth of invasion [11,25]. The Spetzler–Martin grading system [23] is widely used for cerebral AVMs, assessing complexity based on size, location, and venous drainage.

Several authors emphasize how early intervention, especially for lesions at Schobinger stage II, can prevent irreversible soft tissue and functional damage. Our findings further support this perspective, showing that patients treated before significant progression experienced fewer complications and recurrences.

AVMs represent 10% of vascular malformations and are currently classified according to the International Society for the Study of Vascular Anomalies (ISS-VA). This classification distinguishes four types of vascular malformations: (1) simple vascular malformations, (2) capillary malformations (CMs), (3) lymphatic malformations, and (4) venous malformations [8]. The ISS-VA classification is instrumental in determining complication risks and treatment strategies.

Recent clinical reports suggest that embolization strategies incorporating outflow occlusion yield improved nidus control, especially in high-flow lesions with dominant draining veins.

AVMs are often asymptomatic but can increase in volume and size, alter hemodynamics, and become symptomatic following specific stimuli [26]. Their expansion can be triggered by mechanical factors (trauma, infection, iatrogenic causes such as biopsies, surgery, or embolization) or hormonal changes (puberty, pregnancy, hormone therapy), although the precise pathogenesis remains incompletely understood [27]. While hormonal changes are considered potential triggers for AVM growth, Liu et al. did not observe progression in pregnant women with quiescent lesions [28].

When symptomatic, AVMs can cause severe complications, including heart failure, neuropathy, pain, and bleeding [8]. Current treatment options include surgery, endovascular therapy, or stereotactic radiosurgery, aimed at resecting, embolizing, or irradiating the lesion [29,30,31]. Despite therapeutic advancements, AVMs remain challenging to manage due to a high recurrence rate of approximately 25% within the first year post-intervention, regardless of the treatment approach [8,28,32].

It is essential to distinguish between technical success and true clinical resolution. While complete angiographic occlusion was achieved, sustained symptom relief and nidus stability over time are the ultimate metrics of success.

Diffuse lesions with multiple feeding vessels further complicate successful treatment, necessitating close follow-up and repeated interventions [28].

This suggests that anatomical accessibility plays a crucial role in determining treatment modality, and future protocols might benefit from pre-procedural imaging strategies that optimize venous access planning.

Surgical resection, while a potential treatment option, has significant drawbacks, including a high risk of massive intraoperative hemorrhage, ischemia-induced neovascularization leading to recurrence, and difficulties in achieving radical resection. Therefore, surgery is considered a first-line treatment only when complete removal of the AVM is feasible [33]. Targeted medical therapy can be used alone or in combination with invasive treatments to reduce the proliferative potential of AVMs, particularly in progressive stages. Several molecules targeting different pathways are under investigation, including PI3KCA (Alpelisib), mTOR (Sirolimus), MEK (Trametinib), BRAF (Dabrafenib), and VEGF (Bevacizumab); however, currently, no conclusive evidence from large-scale prospective studies supports their efficacy [33,34,35,36].

Collaboration with dermatology, vascular surgery, and medical genetics may expand therapeutic options, particularly in syndromic or refractory cases.

**Figure 4:**
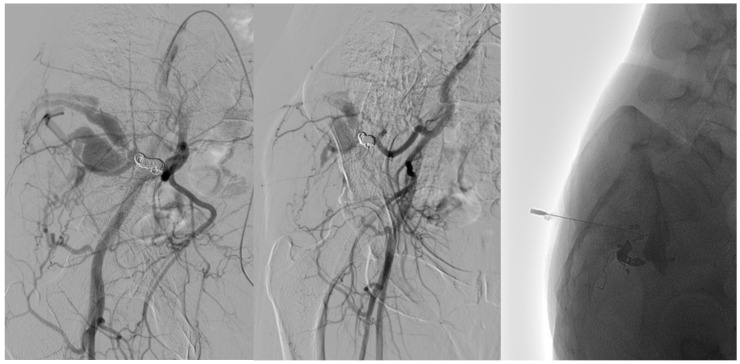
Following a 6-month ultrasound follow-up, there was evidence of recurrence from the collateral branch of the gluteal artery. **The left image** shows an angiographic examination performed pre-procedure after super-selective catheterization using a Progreat microcatheter. The **center image** shows post-embolization angiography performed by EVOH. The venous component was treated in the same session by direct puncture by injecting Atossisclerol.

In recent years, treatment techniques have evolved, with trans-arterial embolization emerging as a preferred approach due to its lower complication rates and reduced recurrence [2,13,19,20,27,29,30]. However, recurrence remains a significant challenge, occurring in 25% of patients within the first year post-treatment and potentially up to 10 years later. Therefore, long-term follow-up is essential to detect recurrences. In our study, a single embolization session was sufficient to resolve the condition, with the highest success rate achieved through combined arterial and venous treatment. Specifically, the arterial side was treated using Onyx, PVA, or coils, while the venous side was treated via direct puncture with Lauromacrogol [Figure 4]. This combined approach was associated with a low recurrence rate (0%).

This study has several limitations. The small sample size limits the statistical power of our comparisons and precludes definitive conclusions. The retrospective design may introduce selection or reporting bias. Furthermore, the follow-up duration, though consistent with other series, may not capture late recurrences that could emerge beyond 6 months.

Another limitation of our study is the heterogeneity of the two treatment groups regarding AVM location. Certain AVMs, such as those in the limbs, were more accessible for direct puncture due to their anatomical location, facilitating venous-side treatment. In other cases, only the arterial side was treated. In patients with a significant arterial component, a two-stage or, in some cases, three-stage intervention was pre-planned to avoid excessive blood loss in a single procedure and to minimize ischemic complications.

This additional session was not considered a recurrence for patients who had a second planned procedure as part of the initial treatment strategy. The recurrence rate was determined only after all planned treatments were completed.

In conclusion, our experience supports the feasibility, safety, and potential clinical advantage of a combined endovascular approach in treating complex extracranial AVMs. While our findings require confirmation in larger prospective studies, they provide preliminary evidence that dual inflow–outflow embolization may improve treatment durability in select patients with high-flow AVMs.

## Figures and Tables

**Table 1:** Comparison of patient characteristics, treatment strategies, and outcomes between Type I and Type IV AVMs. The table highlights differences in embolic material selection, anatomical distribution, and recurrence rates. No statistically significant differences were observed between the two groups in terms of recurrence (*p* = 0.50) or complications (*p* = 1.00).

Variable	Type I (n = 9)	Type IV (n = 5)	*p*-Value
Sex (male)	3/9 (33.3%)	2/5 (40%)	1.00
Age (years, mean ± SD)	34.14 ± 22.66	27.75 ± 22.44	0.66
Location			
Lower limb	5 (55.6%)	5 (100%)	0.22
Neck	2 (22.2%)	0	0.50
Pelvic	1 (11.1%)	0	1.00
Upper limb	1 (11.1%)	0	1.00
Embolic Material for the Arterial Side			
Coils	3 (33.3%)	1 (20%)	-
Coils + PVA	4 (44.4%)	0	-
Coils + EVOH	0	1 (20%)	-
EVOH	1 (11.1%)	1 (20%)	-
PVA	0	1 (20%)	-
EVOH + PVA	1 (11.1%)	0	-
Treatment Approach			
One-stage	5 (55.6%)	3 (60%)	1.00
Two-stages	4 (44.4%)	2 (40%)	1.00
Outcomes			
Recurrence	2 (22.2%)	0	0.50
Complications	1 (11.1%)	0	1.00

**Table 2:** Comparison of single-stage versus multi-stage embolization procedures. This table outlines the distribution of AVM types, anatomical locations, access sites, and embolic agents used in both groups. Recurrence was observed in 12.5% of single-stage procedures and 16.7% of multi-stage procedures.

Variable	Single Attempt (n = 8)	Two or More Attempts (n = 6)
AVM Type		
Type I	5	4
Type IV	3	2
Location		
Lower limb	5	5
Neck	1	1
Upper limb	1	0
Pelvis	1	0
Access Site		
Arterial	5	4
Arterial + Venous direct access	3	2
Embolic Material		
EVOH	1	1
Coils + PVA	2	2
Coils	2	1
Coils + Lauromacrogol	1	1
PVA + Lauromacrogol	1	0
Recurrence	1/8 (12.5%)	1/6 (16.7%)

## Data Availability

The original contributions presented in this study are included in the article. Further inquiries can be directed to the corresponding authors.
